# Electrolyte Disturbances Related to Sodium and Potassium and Electroconvulsive Therapy: A Systematic Review

**DOI:** 10.3390/jcm12206677

**Published:** 2023-10-22

**Authors:** Adam Gędek, Michał Materna, Paweł Majewski, Anna Z. Antosik, Monika Dominiak

**Affiliations:** 1 Department of Pharmacology, Institute of Psychiatry and Neurology, Sobieskiego 9, 02-957 Warsaw, Poland; 2 Praski Hospital, Aleja Solidarności 67, 03-401 Warsaw, Poland; 3 Bielanski Hospital, Cegłowska 80, 01-809 Warsaw, Poland; 4 Stefan Cardinal Wyszynski Regional Specialist Hospital, Aleja Kraśnicka 100, 20-718 Lublin, Poland; 5 Department of Psychiatry, Faculty of Medicine, Collegium Medicum, Cardinal Wyszynski University in Warsaw, Woycickiego 1/3, 01-938 Warsaw, Poland

**Keywords:** hyponatremia, hypernatremia, hypokalemia, hyperkalemia, ECT

## Abstract

Introduction: Electrolyte disturbances related to sodium and potassium affect patients with mental disorders undergoing electroconvulsive therapy (ECT). The objective of this study was to systematically summarize the data regarding ECT and electrolyte disturbances related to sodium and potassium. Materials and methods: A systematic literature review in accordance with PRISMA guidelines was conducted. Clinical studies of patients receiving ECT with electrolyte disturbances reported before or after treatment were included. Results: We identified nine case reports and two retrospective studies describing electrolyte abnormalities occurring before or after ECT. ECT was effective and safe in patients with hyponatremia and hypernatremia, including the elderly patient population. This treatment was also effective in treating psychiatric symptoms that may persist after ionic equalization. Electrolyte disturbances after ECT were rare. Reports have suggested that succinylcholine used as a muscle relaxant was the main cause of hyperkalemia after ECT. Conclusions: Electrolyte control is a crucial aspect of guiding ECT therapy. In the context of sodium-related disorders, it is critical to control patient hydration as part of therapy. In addition, succinylcholine should not be used in patients with immobilization, such as catatonia or neuroleptic malignant syndrome. It is necessary to conduct further studies to clarify whether electrolyte concentration affects ECT parameters and clinical efficacy. In addition, it is necessary to assess the influence of various anesthetics on these conditions during ECT. The result of this review should be interpreted bearing in mind the small number of studies conducted to date and the low quality of the evidence they provide.

## 1. Introduction

Sodium and potassium are two main ions in the human body that affect homeostasis and metabolism. As a result, biochemical and enzymatic reactions affect the structure and function of cell membranes, neurotransmission, nerve signal conduction, and muscle contraction [1]. Electrolyte disturbances are a consequence of excess or deficiency of serum ions, leading to systemic symptoms of varying severity and rate of progression [2]. Consequently, they contribute to higher morbidity and mortality rates [3]. The most common ones are sodium-related electrolyte disturbances (hyponatremia and hypernatremia), and potassium-related abnormalities (hypokalemia and hyperkalemia). Electrolyte disturbances occur frequently among hospitalized patients [4] including those in psychiatry wards [5,6]. The prevalence of these abnormalities is higher in patients with severe general health conditions [7]. Furthermore, they are common side effects of psychiatric medications, especially selective serotonin reuptake inhibitors (SSRIs) [8,9].

Electroconvulsive therapy (ECT) is an effective and safe treatment for severe psychiatric disorders [10]. In this procedure, the patient is anesthetized, and their muscles are relaxed using premedication drugs, short-acting anesthetics, and relaxants. An electrical stimulus transmitted through special electrodes, located unilateral or bilateral, can therefore be used in a safe and controlled setting to induce seizure activity. A serum sodium and potassium concentration assessment is a part of the preparation for treatment [11]. The most common ionic imbalances can thus be corrected before treatment, ensuring the safety of the procedure. Otherwise, there is a risk of serious adverse events during ECT. A severe sodium imbalance can lead to spontaneous seizures or prolonged epileptic seizures and status epilepticus [12,13]. There are several manifestations of potassium concentration disturbances, including cardiac arrythmia [14]. This is especially relevant during ECT, in which electrical stimulus activates the autonomic nervous system and influences heart rate or blood pressure [15]. Severe electrolyte abnormalities are therefore usually caught and compensated before the procedure. However, it is unclear how to manage in the case of chronic disturbances (e.g., inadequate vasopressin secretion syndrome—SIADH or other electrolyte disturbances), whether the ECT procedure is still safe and effective.

Another important point is to know when electrolyte disturbances may occur after ECT. Although not common, this complication can have life-threatening consequences. It is important to keep in mind that the entire procedure does not just involve the flow of electrical stimulus, but also the premedication, anesthesia, and relaxation drugs. Drugs used to induce anesthesia or muscle relaxation affect potassium concentration in the blood [16]. In some studies, succinylcholine has been linked to an increase in serum potassium levels during ECT [17,18,19,20]. Usually, ECT treatments are safe, and significant electrolyte disturbances have not been reported, however, there are cases when they can disrupt the patient’s electrolyte balance. A risk group can be identified, and aspects of treatment management can be determined by identifying these factors. Table 1 provides information on laboratory standards based on the severity of these conditions and how they are affected by the main anesthetics used during ECT [21,22,23,24,25,26,27,28,29,30,31,32,33,34] (Table 1).

As ECT is used as a treatment for severe conditions with often coexisting chronic electrolyte disturbances that cannot be entirely corrected, the question arises whether such treatment may be still considered as a safe and effective in this population. Moreover, it is unclear whether the ECT procedure affects ionic abnormalities. As we found no paper in the literature dealing with this problem, the linkage between electrolyte disturbances and ECT seems to remain unclear.

Thus, the aim of this paper was to systematically review and analyze the data from clinical studies regarding electroconvulsive therapy and electrolyte disturbances related to sodium and potassium. Specifically, we aimed at answering two main questions: (1) is ECT safe and effective in patients with electrolyte disturbances? and (2) can ECT procedure induce electrolyte disturbances?

## 2. Materials and Methods

This systematic review was performed in accordance with the PRISMA (Preferred Reporting Items for Systematic Reviews and Meta-Analyses) statement and based on the PICO format (patients, interventions, comparison, outcome) [35,36].

### 2.1. Information Sources, Search Strategy, Selection Process

On 5 March 2023, two reviewers performed independently a search of PubMed, Web of Science, and Scopus, without using any filters and limits. Only well-established databases with a large number of articles and citations in the medical field accessible to reviewers through the institution were selected. The search strategy utilized was as follows for each database: (“electroconvulsive therapy” OR “ect.”) AND (“electrolytes disturbances” OR “hyponatremia” OR “hypernatremia” OR “sodium” OR “hypokalemia” OR “hyperkalemia” OR “potassium”). Search results were downloaded onto the Mendeley Desktop and managed with this application. After filtering the duplicates, two independent researchers screened titles and abstracts selecting papers for full-text assessment. Additionally, two independent researchers performed a search in the clinical trial registry and conducted backward and forward citation chaining. Any disagreement was resolved by consultation with a third reviewer.

### 2.2. Data Collection Process, Data Items

The following data were retrieved manually in the specially created form: first author, year of publication, study design, number of patients, age, gender, type of electrolyte disturbances (defined as: hyponatremia—sodium concentration < 134 mEq/L, hypernatremia—sodium concentration > 145 mEq/L, hypokalemia—potassium concentration < 3.5 mEq/L, hyperkalemia—potassium concentration > 5.5 mEq/L) and measurement before or after ECT, clinical picture (psychiatric diagnosis, treatment before ECT, other conditions), ECT characteristics (electrode placement, anesthetic drugs, number of treatments), and outcomes (efficacy—impact on symptoms assessed on a clinical scale or narrative described; safety—reported adverse effects and complications, impact on electrolyte disturbances—change in blood concentration).

### 2.3. Eligibility Criteria

A PICO framework question was developed to identify suitable papers for inclusion in the review: Patients—patients with electrolyte disturbances related to sodium and potassium before or after ECT treatment; Intervention—electroconvulsive therapy; Comparison—due to limited studies comparing ECT to other modalities we included studies on ECT alone; Outcomes—clinical symptoms, electrolytes concentration. Based on this, eligibility criteria were developed. The inclusion criteria were (1) patients receiving ECT; (2) electrolyte disturbances reported before or after ECT treatment; (3) clinical study of any design. The exclusion criteria were (1) not in English; (2) not original study: review, comment, letter, editorial, animal study, conference note; (3) not published; (4) not report required outcomes; (5) not conform with PICO.

### 2.4. Study Risk of Bias Assessment

The Joanna Briggs Institute (JBI) Critical Appraisal Checklist for case reports was used for appraising the quality of the included articles [37]. This checklist consists of an 8-item scale that includes the patient’s demographic characteristics, medical history, current clinical condition, description of diagnostic tests, treatment, post-intervention clinical condition, adverse events, and the provision of a takeaway lesson. After investigating several rating systems for the evaluation of the quality of observational studies (such as the Newcastle−Ottawa System or the Risk of Bias Assessment Tool for Non-randomized Studies), we found them inappropriate for this review. We developed a rating tool based on the tools quoted above yet tailored for the aims of this review. The score ranged from 1 to 6 points. One point was given for the following criterion: (1) sample size > 100; (2) representativeness of the sample; (3) possible confounders reported in detail (demographic and clinical characteristics of participants); (4) an adequate and detailed description of the ETC procedure (indication the location of the electrodes and the anesthetics used); (5) the quality of measurement methods (i.e., description of the time and method of collection laboratory tests, assessment tool for clinical symptoms); (6) comparative study (the aim of the study was to compare two groups of patients, one of whom had electrolyte disturbances). Each article was scored independently by two reviewers. A third reviewer was consulted for opinion in case of disagreement.

### 2.5. Synthesis Method

As a result of the limited evidence and heterogeneity of the studies, a narrative synthesis of evidence was prepared for each review objective and outcome.

## 3. Results

### 3.1. Study Selection and Characteristics

**Figure 1:**
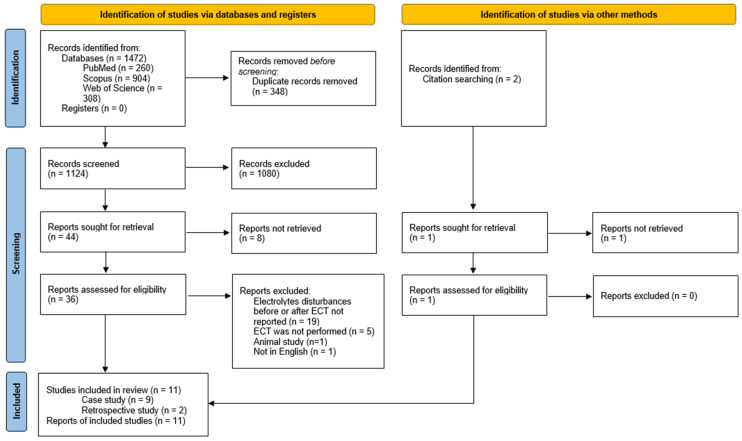
Selection process—PRISMA flowchart.

Literature was searched and 1472 papers were found. A total of 37 articles were screened as per the eligibility criteria, and finally, 9 case reports [38,39,40,41,42,43,44,45,46] and 2 retrospective studies were included [47,48]. The PRISMA flowchart is presented in Figure 1. One study was included based on title and abstract screening, however full-version was not retrieval [49].

### 3.2. Risk of Bias in Studies

Table 2 displays the risk of bias that has been evaluated with the help of the Critical Appraisal Checklist for Case Reports [37]. Table 3 displays the risk of bias that has been evaluated for retrospective study [47,48]. All studies were included in narrative synthesis in this systematic review.

### 3.3. Results of Individual Studies and Syntheses

#### 3.3.1. General Characteristic

In our systematic review, 9 case reports [38,39,40,41,42,43,44,45,46] and 2 retrospective studies from 1989–2020 were included [47,48]. Further characterization and analysis of the studies were carried out in accordance with the previously adopted division on electrolyte disturbances occurring before or after ECT. One case report was eligible for both sections, as it described hyponatremia that was present before the procedure, but also appeared after ECT, despite previous compensations [44]. One retrospective study was considered for inclusion in the two sections since it included two samples of patients, which measured electrolytes before and after ECT, respectively. However, it has been included only in “Electrolytes disturbances after ECT” section due to the lack of reporting of outcomes, which we are interested in pre-ECT patients [47].

#### 3.3.2. ECT for Patients with Electrolytes Disturbances

##### Characteristic of Included Studies

Studies from 1990–2020 were included in the review of patients with electrolyte abnormalities who received ECT. Five case reports [38,41,44,45,46] and one retrospective study [48] were identified. Hyponatremia was reported in four case studies, where patients ranged in age from 48 to 83 years old [41,44,45,46], and hypernatremia in one patient at age 52 years old [38]. Three of the patients were depressed [38,44,46], one was depressed with comorbid obsessive-compulsive disorder (OCD) [41], and one was catatonic secondary to hyponatremia [45]. One retrospective study included 207 patients with a mean age of 55.6 (SD 18.9) years [48]. In this group, 12 patients had hyponatremia and 4 hypernatremia. Different ECT procedures were used in all of these studies (Table 4). There were no studies conducted on patients receiving ECT reporting on hyperkalemia or hypokalemia.

##### Efficacy of Electroconvulsive Therapy for Patients with Electrolyte Disturbances

Three case studies documented successful electroconvulsive procedures in patients with hyponatremia (117–132 mmol/L) [41,44,46]. SIADH was the cause of hyponatremia in one case [46] and polydipsia in the other one [44]. Improvements in hyponatremia after ECT treatments were reported in both studies. ECT treatment was also administrated to a patient with chronic hyponatremia in another study [41]. The patient’s mental state improved after ECT and hyponatremia remained stable after this treatment [41]. All three cases of patients with hyponatremia treated with ECT described above concerned older people (>65 years of age) [41,44,46].

In the course of sodium-related disorders in two of included studies, patients developed catatonia [38,45]. In one case, catatonia caused by hyponatremia (109–120 mmol/L) possibly due to adrenal insufficiency was reported [45]. After electrolyte supplementation, steroids, and a trial of lorazepam, electrolyte disturbance was managed, but catatonia symptoms did not improve. A reduction in symptoms was observed after ECT therapy [45]. In another case, a patient suffering from depression developed hypernatremia. At the same time stupor was observed. While electrolyte abnormalities were successfully compensated, only ECT treatment improved clinical symptoms. In this study authors suggest that the psychogenic factor was considered to contribute to the presence of stupor [38].

##### Safety of Electroconvulsive Therapy for Patients with Electrolytes Disturbances

In the case report studies, all ECT treatments were safe in patients with hyponatremia [41,44,45,46]. In two studies involving patients with hyponatremia an equalization in sodium level after ECT was reported. ECT also proved safe in a patients with hypernatremia and catatonia [38]. According to one retrospective study, four patients with hypernatremia and twelve with hyponatremia received safe treatment [48]. The included studies did not report any prolonged seizures or other serious adverse events.

#### 3.3.3. Electrolytes Disturbances after ECT

##### Characteristic of Included Studies

Studies from 1989–2014 were included in the review of patients with electrolyte abnormalities after ECT treatment. Five case reports [39,40,42,43,45] and one retrospective study [47] were identified. Hyperkalemia was reported in 3 case studies. Hypokalemia was reported in two case studies. The age of patients ranged from 34 to 55 years old. Among the patients, one was depressed [40], one had neuroleptic malignant syndrome (NMS) [39], one was non-psychiatric with catatonia secondary to hyponatremia [45], and two had catatonia in the course of schizophrenia [42,43]. One retrospective study included 562 patients with a mean age of 54.6 (SD 19.3) with hyponatremia, hypernatremia, hypokalemia, and hyperkalemia [47]. Different ECT procedures were used in all of these studies (Table 5).

##### Efficacy

In four case reports ECT treatment was highly effective on clinical symptoms in patients with depression and agitation, catatonia in the course of schizophrenia, catatonia secondary to hyponatremia, and neuroleptic malignant syndrome [39,40,43,45]. One study did not report clinical efficacy [42].

##### Safety

Finlayson et al. [40] described a case of spontaneous convulsions and hyponatremia after 10 ECT treatments in a depressed patient. However, the patient had polydipsia and drank between 5–10 L of water per day during hospitalization and ECT procedures. Grover et al. [45] described a case of hyponatremia that was present before after third ECT treatments in catatonic patient. The catatonia was secondary to hyponatremia (possibly due to adrenal insufficiency), however the ECT treatments were safe, no adverse events were reported.

We identified three case reports of hyperkalemia after electroconvulsive therapy. In two cases, ECT treatments caused hyperkalemia in patients with schizophrenia and catatonia. Symptoms occurred after succinylcholine (120–140 mg) was administered and resolved after switching to another medication (mivacurium, atracurium) [42,43]. Similar observations were made for a patient with the neuroleptic malignant syndrome. Succinylcholine was administered in a dose of 1.2 mg kg^−1^ and hyperkalemia was observed after four treatments. When rocuronium (1.2 mg kg^−1^) was used instead, it was not reported [39].

Important information was provided by one retrospective study included in this review [47]. Electrolyte abnormalities related to sodium and potassium were not different between patients who received ECT and those who did not. However, an abnormal sodium level after ECT predicted hospital readmission [47].

## 4. Discussion

Electroconvulsive therapy has for years remained one of the most effective and safest treatments in psychiatry. Although there are no strict contraindications, several tests, including sodium and potassium measurements are performed prior to treatment to ensure maximum safety for patients. This review systematically evaluates the available studies linking ECT therapy with electrolyte disturbances related to sodium and potassium before and after treatment.

The first conclusion that emerges from this review is the limited number and value of evidence from studies on electrolyte disturbances and ECT. This can be partly explained by the ability to compensate for sodium and potassium levels before treatment, which in most cases is not a problem. According to Lafferty et al., electrolyte disturbances before ECT were relatively rare, affecting 5% of patients. However, the author suggested the measurement of sodium level is useful, as is measurement of potassium levels because of potential cardiac compilation [47]. When analyzing the results, we have also noticed that ECT procedures were not reported properly.

Based on the studies we identified, electroconvulsive therapy can be effective and safe in patients suffering from sodium electrolyte disturbances. It is estimated that 5–11% of patients in psychiatric wards suffer from hyponatremia [50,51]. Various factors can affect it, including aging, uncontrolled fluid intake, comorbidities, and psychiatric medication, especially SSRIs [52]. There is a wide range of clinical manifestations depending on the severity of the condition [53]. Hypernatremia, which is mainly associated with dehydration, is less common, but it is also present [54]. An essential part of the treatment process is monitoring the water-electrolyte balance. Normally, electroconvulsive therapy should begin after any abnormalities have been equalized; however, sometimes this is not possible based on the patient’s condition or other factors. Literature data, however of limited level of evidence, indicate that electroconvulsive treatment is safe and effective for patients suffering from hyponatremia or hypernatremia in such situations [41,44,46,48,49].

Hyponatremia is also a common side effect of drugs used to treat mental disorders [55,56]. ECT appears safer than pharmacotherapy in this respect. Only two studies reported hyponatremia after ECT treatment. In one case the patient drank many liters of water [40], in second the patient had hyponatremia before treatment [45]. This context implies the importance of controlling hydration during patients’ stay in the ward as well as their preparation for treatment. A clinical symptom that should draw attention is polydipsia or polyuria. Furthermore, electrolyte concentrations should be monitored regularly, especially in psychiatric departments. Thus ECT seems to remain a safe alternative to consider not only in cases of drug-resistant but also among patients who have experienced electrolyte disturbances during pharmacotherapy [10]. There is also a case in the literature demonstrating the value of biological treatments and the safe use of rTMS (repetitive Transcranial Magnetic Stimulation) in a patient with depression and antidepressant-associated hyponatremic seizures [57].

In patients with hyponatremia, ECT therapy has been demonstrated to be safe, as well as effective. There is evidence that sodium levels may affect therapeutic efficacy. In one sense, it may affect the seizure threshold, which determines the effectiveness of treatment. It has been demonstrated that the risk of seizures increases with a decrease in serum sodium concentration [13]. However, serum sodium did not correlate with seizure length or seizure threshold according to Rasmussen et al. [48]. On the other hand, sodium levels may increase interhemispheric coherence (IHC), which may help to generalize seizures during ECT. According to Belz et al., a 1 mmol/L reduction in serum sodium concentration increased IHC by 0.678% [58]. To clarify whether serum sodium levels are related to ECT effectiveness, further studies on larger patient populations, preferably prospective, evaluating electrolyte levels before, during, and after ECT treatment are needed. If such firm evidence existed, it would seem that a simple and accessible solution would be to support ECT by ensuring adequate and controlled hydration of the patient.

Monitoring serum potassium levels is also a crucial part of monitoring of ECT therapy. Nonetheless, there are serious conduction disturbances associated with these conditions, and drugs administered during ECT may also affect them. It has been reported that hypokalemia and hyperkalemia occurred after ECT with the same frequency as in patients who were treated with medications [47]. However, these complications can be extremely dangerous. The potassium level has been evaluated in several studies before and after ECT for safety, but not for treatment efficacy. There have been reports in literature, that succinylcholine when used as a relaxant, increased potassium concentration after the treatments, but not beyond normal levels [17,18,19,20]. This is probably related to the synchronous contraction of muscles containing blood with increased potassium concentration following succinylcholine [17]. According to Aaron et al., this increase was similar for the 0.5 mg/kg and 1 mg/kg doses, but the higher dose was associated with an improved modification of convulsions with comparable hemodynamics and side effects [18]. In our review, we identified three cases of hyperkalemia after ECT [39,42,43]. Succinylcholine was used in each case. In light of the fact that it is one of the most common drugs used to relax the muscles during electroconvulsive therapy, this evidence is substantial. It is therefore crucial to understand high-risk conditions in order to ensure safe treatment. The concentration of potassium may also be affected by a number of factors, including anxiety, medication, CO_2_, or anesthesia drugs [16,59], as well as immobilization [60], which is particularly relevant to patients with catatonia, NMS or neurological injury. Regardless of the condition, hyperkalemia can develop after treatment even when potassium levels were normal before ECT. Succinylcholine should be used with extreme caution in conditions involving severe psychiatric or somatic symptoms accompanied by immobility or other risk factors for hyperkalemia. Other muscle relaxants should be considered when baseline potassium levels are high or other risk factors for hyperkalemia exist. Cistacurium raised potassium levels less than succinylcholine in a recent study, suggesting an alternative for these patients [16]. A further finding of our review is that it can be difficult to differentiate NMS from catatonia in clinical practice. We included two cases in which catatonic patients were initially treated for NMS [42,43]. Consequently, the treatment time for catatonia may be delayed and the immobilization period may be prolonged, which increases the risk of hyperkalemia.

Electrolyte abnormalities are particularly common in older people, in whom they are persistent or recurrent. The use of ECT in older populations has also been recognized as a safe and effective treatment option [61]. A higher age is also a predictor of a better response [10]. It may, however, cause adverse effects, especially when combined with somatic disorders. As a result of comorbidities, hydration problems, or polypharmacy, electrolyte disturbances are more common in this population. However, the case reports we identified suggest that even in hyponatremia, ECT is effective and safe in this group of patients [41,44,46,49]. In this population, this treatment modality may even be more effective than others, according to existing literature [61]. Given that ECT is increasingly being performed in elderly patient populations, more studies with higher levels of evidence (observational studies, RCTs) are needed in order to improve the safety of ECT treatment in this population. Although the aim should always be to compensate for electrolyte disturbances, the question of whether ECT should even be considered for uncompensated hyponatremia in elderly patients remains to be resolved.

Electroconvulsive therapy can also be an effective treatment for symptoms that occur secondary to electrolyte disturbances, such as catatonia. According to identified studies, ECT was applied to two patients whose electrolyte abnormalities had been successfully corrected before treatment, but whose clinical symptoms continued until they received ECT [38,45]. However, the relationship between electrolyte disturbances and catatonia remains unclear. It is possible that hyponatremia overlapped with the psychogenic symptoms, which would explain the effectiveness of ECT in these case reports. Additionally, in one case report ECT was effective in patients with hyponatremia and SIADH. The treatment was safety, but also the resolution of SIADH symptoms was observed. The second similar case report exists in the literature, but this report was not retrieved [49]. This evidence indicates that ECT should be considered as a potential therapeutic option in patients with SIADH.

While our review has highlighted the potential safety of ECT in patients with electrolyte disturbances, it is essential to acknowledge the factors associated with limiting its application. Elevated intracranial pressure is only one absolute contraindication of this treatment, while relative contraindications include acute somatic conditions [62]. However, as a medical procedure, ECT is not without potential adverse effects. The most common are self-limited and can be managed symptomatically, including headache, nausea, myalgia, and confusion [63]. The safety and tolerability of ECT have often been questioned especially due to the associated cognitive side effects (i.e., disorientation, anterograde amnesia, and retrograde amnesia, memory impairment) [64]. These adverse effects are common but usually improve within a few weeks; however, memory impairment may rarely persist [63]. To date, various modifications, such as electrode placement have been developed to help minimize cognitive side effects [65].

### Limitations

As a first limitation, only a small amount of material was identified and included in the study. Furthermore, only case reports and retrospective studies were identified. Therefore, the quality of the evidence is low, and the results should be interpreted with caution. To overcome these limitations, more studies are needed, especially prospective clinical trials evaluating ECT’s efficacy and safety in patients with electrolyte disturbances before and after therapy. Moreover, most of the studies did not report exact treatment parameters, such as stimulus length, amount of current [39,42,43,46,47], or at least electrode placement [39,42,43,47]. Further, some papers lack information about the anesthetics used, which affect electrolyte concentrations after therapy in an important, perhaps crucial way. It is recommended that clinical trials should report in detail the ECT intervention using appropriate tool such as TIDieR (Template for Intervention Description and Replication) in order to obtain more reliable results [66]. Recently, a dedicated version of this tool has been developed for another biological treatment method—rTMS [67]. In the context of ECT, it would allow features such as electrode location, anesthetics used, number of treatments, and seizure quality measurements to be considered in the evaluation of this treatment. By comparing treatment parameters, it will be possible to determine which settings are most favorable and safe. Furthermore, incomplete reporting can limit the implementation of ECT in clinical practice and complicate the precise interpretation and comparison of study results.

## 5. Conclusions

Monitoring electrolyte serum levels is an integral part of ECT therapy. According to limited evidence from case reports and retrospective studies, the presence of mild hyponatremia or hypernatremia should not be considered a contraindication to treatment when the clinical situation warrants it. Moreover, electrolyte abnormalities are particularly common in older people, in whom they are persistent or recurrent. Given that ECT is increasingly being performed in the elderly patient population, more studies with a higher level of evidence (preferably prospective observational studies) are needed in order to improve the safety of ECT treatment in this population. Monitoring hydration, which affects serum sodium levels, is essential to guiding treatment. Patients with baseline hyperkalemia that has been compensated for and those with risk factors for hyperkalemia should be closely observed after each ECT treatment. The use of a drug other than succinylcholine for muscle relaxation is recommended in this situation. In regard to the efficacy of ECT and electrolyte disturbances, there is currently a lack of research on this topic; prospective studies on a large population would be needed. The safety, efficacy, and electrolyte disturbances of anesthetics used during treatment could also be evaluated in further studies, especially in a group of high-risk patients, including those immobilized by catatonia, NMS, or neurological injury.

## Figures and Tables

**Table 1:** Degrees of laboratory electrolyte disturbances related to sodium and potassium and potential influence of premedication drugs, short-acting anesthetics, or relaxants used during ECT on these conditions.

Electrolytes Disturbances	Degrees of Severity			Drug Used during ECT
	Mild (mEq/L)	Moderate (mEq/L)	Severe (mEq/L)	
Hyponatremia	130–134	125–129	<125	Not reported
Hypernatremia	145–149	150–169	>170	Ketamine [21,22,23], Thiopental (false hypernatremia) [24,25]
Hypokalemia	3.5–2.5	2.5–3.0	<2.5	Atropine [26,27], Propofol [28,29,30], Thiopental [28,29,30]
Hyperkalemia	5.5–6.0	6.0–7.0	>7.0	Succinylcholine [31,32,33,34], Propofol [28,29,30], Thiopental [28,29,30]

**Table 2:** Critical appraisal of case reports included in this review.

Author (Year)	Domain 1	Domain 2	Domain 3	Domain 4	Domain 5	Domain 6	Domain 7	Domain 8	Overall
MacMillan (1990) [46]	yes	yes	yes	yes	unclear	yes	yes	yes	7/8
Greer and Stewart (1993) [44]	yes	unclear	yes	unclear	yes	no	no	yes	4/8
Mashimo (1996) [38]	yes	yes	yes	yes	yes	yes	unclear	yes	7/8
Grover (2012) [45]	yes	yes	yes	yes	unclear	yes	unclear	yes	7/8
Kern (2020) [41]	yes	unclear	unclear	unclear	yes	yes	yes	no	4/8
Finlayson (1989) [40]	yes	yes	yes	yes	unclear	yes	yes	yes	7/8
Cooper (1999) [42]	yes	yes	yes	yes	unclear	unclear	yes	yes	6/8
Hudcova (2006) [43]	yes	unclear	yes	yes	unclear	yes	yes	yes	6/8
Koster (2014) [39]	yes	yes	yes	yes	unclear	yes	yes	yes	7/8

**Table 3:** Quality assessment of retrospective studies included in this review.

Author (Year)	Domain 1	Domain 2	Domain 3	Domain 4	Domain 5	Domain 6	Overall
Lafferty (2001) [47]	1	1	1	0	1	0	4/6
Rasmussen (2007) [48]	1	1	1	1	1	0	5/6

**Table 4:** Studies included patients with electrolyte disturbances treated with ECT.

Author (Year)	Study Design	Number of Patients/Age/Gender (M/F)	Electrolyte Disturbance: (1) Type of Disturbance (2) Measurements before Treatment	Clinical Picture: (1) Psychiatric Diagnosis (2) Treatment before ECT (3) Other Conditions	ECT Characteristics: (1) Electrode Placement (2) Anesthetics Drugs (3) Number of Treatments	Outcomes: (1) ECT Influence on Clinical Symptoms (2) Electrolyte Levels after ECT
MacMillan et al. (1990) [46]	Case report	1/72/M	(1) Hyponatremia (2) ECT was administered when serum sodium amounted to 130 mmol/L; on admission it was 127 mmol/L, the lowest value during hospitalization was 120 mmol/L	(1) Treatment resistant depression—major depressive episode (2) Doxepin, nortriptyline, tranylcypromine, alprazolam (3) Syndrome of inappropriate antidiuretic hormone secretion (SIADH), Chronic obstructive lung disease	(1) Unilateral right ECT (2) No info (3) 12	(1) The improvement in clinical symptoms was observed after 12 ECT treatment; ECT treatment was safe (2) In following tests, with unrestricted fluid intake, serum sodium did not fall below 135 mmol/L
Greer and Stewart (1993) [44]	Case report	1/83/F	(1) Hyponatremia (2) On admission serum sodium amounted to 121 mmol/L; during hospitalization it range between 117–128 mmol/L	(1) Treatment resistant depression (2) Diltiazem, digoxin, lorazepam, propoxyphene napsylate, two trials of antidepressants, demeclocycline (3) Several hip dislocations 1 year previously, coronary artery disease, hypertension, myocardial infraction, drunk large quantities of water	(1) Bilateral ECT (2) methohexital (35 mg), succinylcholine (60 mg), esmolol (80 mg) (3) 5	(1) Data not available (2) Two days after first ECT treatment sodium level increased to 132 mmol/L and 136 mmol/L after fourth ECT treatments;
Kern et al. (2020) [41]	Case report	1/65/F	(1) Hyponatremia (2) On admission serum sodium amounted to 129 mmol/L; during hospitalization it range between 130–132 mmol	(1) Depression, OCD, anorexia (2) Data not available (3) Chronic hyponatremia, Intraparenchymal lateral ventricular cyst (6.3 cm) and periventricular edema	(1) Bilateral ECT (2) Succinylcholine (60 mg), methohexital (50 mg), nitrogliceryne, labetalol (3) 5 in first series; 12 lifetime	(1) The significant improvement in depressive symptoms was observed (2) Hyponatremia remained stable during treatments
Mashimo et al. (1996) [38]	Case report	1/52/F	(1) Hypernatremia (2) On admission serum sodium amounted to 166 mEq/L	(1) Depression and obsessive concern for her handicapped hand, (2) Diazepam, clomipramine (3) Trauma (lost four fingers), dehydration, stupor	(1) Bilateral ECT (2) Amobarbital sodium (200 mg) (3) 6	(1) After six ECT treatments dramatic improvement in stupor symptoms was observed (2) Hypernatremia was corrected before ECT, however clinical symptoms persist; thus the psychogenic factor was considered to contribute to the presence of stupor
Grover et al. (2012) [45]	Case report	1/48/F	(1) Hyponatremia (2) Serum sodium ranged between 109–120 mmol/L with occasional reading of normal sodium level in multiple measurements	(1) Catatonia secondary to hyponatremia (in the background of vomiting and fever) (2) Amisulpiride, iron, hydrocortisone, lorazepam (3) Adrenal insufficiency, anemia	(1) Bilateral ECT (2) Data not available (3) 6	(1) The improvement in catatonic symptoms was observed after 6 ECT treatments. (2) Hyponatremia was corrected before ECT, but clinical symptoms persisted; patient was again found to have hyponatremia after a third ECT, after which she was started on fludrocortisone tablets—after this her sodium level stabilized
Rasmussen et al. (2007) [48]	Retrospective study	207/55.6 (18.9)/59% F	(1) Hyponatremia and hypernatremia (2) Hyponatremia was defined as serum sodium level below 135 mmol/L; hypernatremia was defined as a serum sodium level greater than 145 mmol/L; measurements were performed maximum a week before treatment	(1) ECT treated inpatients	(1) ECT bitemporal (*n* = 84), bifrontal (*n* = 68), right unilateral (*n* = 40) (2) glycopyrrolate, thiopental, remifentanil, etomidate, succinylcholine (3) 207	(1) ECT treatments were safe in all cases of hyponatremia (*n* = 12) and hypernatremia (*n* = 4). Prolonged seizures were not observed.

ECT—electroconvulsive therapy; OCD—obsessive-compulsive disorder.

**Table 5:** Studies including patients with electrolyte disturbances after ECT treatment.

Author	Study Design	Number of Patient/Age/Gender (M/F)	Electrolyte Disturbance: (1) Type of Disturbance (2) Measurements after Treatment	Clinical Picture: (1) Psychiatric Diagnosis (2) Treatment before ECT (3) Other Conditions	ECT Characteristics: (1) Electrode Placement (2) Anesthetics drugs (3) Number of Treatments	Outcomes: (1) ECT Influence on Clinical Symptoms (2) Electrolyte Levels after ECT
Lafferty et al. (2001) [47]	Retrospective study	484/54.1(19.1)/70%F	(1) Hyponatremia, hypernatremia, hypokalemia, hyperkalemia (2) Data not available	(1) Patients received ECT (2) Data not available (3) Data not available	(1) Data not available (2) Data not available (3) Data not available	(1) Abnormal sodium levels were associated with hospital admission after ECT, but without impact on ECT complications. Both re-admissions and complications were not associated with potassium abnormalities. (2) The proportion of Na+ and K+ abnormalities in ECT patients was similar to that in those without ECT.
Finlayson et al. (1989) [40]	Case report	1/55/F	(1) Hyponatremia (2) 106 mmol/L	(1) Depression (2) Trazodone, L-tryptophan, triazolam, flupethixol, thioridazine; past treatment: tricyclic and monoamine oxidase inhibiting antidepressants, neuroleptics, benzodiazepines, lithium (3) Agitation, insomnia, poor appetite, somatic complaints of abdominal burning, loss of concentration and memory, polidypsia	(1) Unilateral right ECT (2) Data not available (3) 10	(1) After eight treatments a decrease in agitation and slight improvement in affect was noted. (2) After 10 ECT treatment patient developed spontaneous seizures with serum sodium 106 mmol/L 50 min after the seizures. She drank 5–10 L of water per day during the hospitalization.
Grover et al. (2012) [45]	Case report	1/48/F	(1) Hyponatremia (2) Data not available	(1) Catatonia secondary to hyponatremia (in the background of vomiting and fever) (2) Amisulpiride, iron, hydrocortisone, lorazepam Adrenal insufficiency, anemia	(1) Bilateral ECT (2) Data not available (3) 6	(1) The improvement in catatonic symptoms was observed after 6 ECT treatments. (2) Hyponatremia was corrected before ECT, but clinical symptoms caused by it remained; patient was again found to have hyponatremia after a third ECT, after which she was started on fludrocortisone tablets—after this her sodium level stabilized
Cooper et al. (1999) [42]	Case report	1/40/F	(1) Hyperkalemia (2) 4.0–6.9 mEq	(1) Catatonia, schizophrenia (2) Amantadine, dantrolene, bromocriptine	(1) Data not available (2) Atropine (0.4 mg), Curare (3 mg), labetalol (10 mg), methohexital (80 mg), succinylcholine (120 mg) (3) 4	(1) Data not available (2) Potassium level was 5.4 mEq after second, 5.5 mEq after third (4.2 before treatment), 6.9 mEq after fourth ECT treatment (4.0 before); the patients was noted to have wide-complex arrythmia after 2nd treatment, and peaking T waves after 4th. Succinylcholine was substitute to mivacurium and potassium level was normal after next treatments.
Hudcova and Schumann (2006) [43]	Case report	1/34/F	(1) Hyperkalemia (2) 6.4 mEq/L	(1) Catatonia, schizophrenia, bipolar disorder (2) Haloperidol, bromocriptine, lorazepam (3) Obesity, fever, pulmonary embolism, intubation and mechanical ventilation	(1) Data not available (2) Succinylcholine (140 mg), etomidate (20 mg), glycopyrrolate (0.4 mg) (3) 3	(1) ECT improved her psychiatric condition (2) Potassium level was 6.4 mEq/L for third ECT treatment and ventricular tachycardia was noted. Then succinylcholine was substitute to atracurium/mivacurium. No electrolyte abnormalities were noted during the following outpatient treatment
Koster et al. (2014) [39]	Case report	1/42/F	(1) Hyperkalemia (2) 11.6 mmol/L^−1^	(1) Neuroleptic malignant syndrome after drug overdose (2) Duloxetine, quetiapine, flurazepam, insulin, activated charcoal	(1) Data not available (2) Succinylcholine (1.2 mg kg^−1^) and etomidate—first 4 sessions; rocuronium (1.2 mg kg^−1^) and etomidate—next 7 sessions (3) 11	(1) In total, patient received 11 ECT sessions with improvement in terms of rigidity, fever and creatine kinase (2) At the four session arterial blood sample showed a potassium of 11.6 mmol/L^−1^ (the patient developed VFib, treated with cardiopulmonary resuscitation; calcium gluconate was administered); in next sessions with rocuronium hyperkalemia was not reported

ECT—electroconvulsive therapy, VFib—ventricular fibrillation.

## Data Availability

The data presented in this study are available upon request from the corresponding author. The data are not publicly available due to privacy or ethical concerns.

## Abbreviations

ECT—electroconvulsive therapy; SSRIs—selective serotonin reuptake inhibitors; SIADH—syndrome of inappropriate antidiuretic hormone secretion; PRISMA—Preferred Reporting Items for Systematic Reviews and Meta-Analyses; PICO—patients/intervention/comparison/outcome; JBI—Joanna Briggs Institute; OCD—obsessive-compulsive disorder; SD—standard deviation; VFib—ventricular fibrillation; rTMS—repetitive Transcranial Magnetic Stimulation; IHC—interhemispheric coherence; NMS—neuroleptic malignant syndrome; RCTs—randomized controlled trials; TIDieR—Template for Intervention Description and Replication; CO_2_—carbon dioxide; Na—sodium; K—potassium.
